# L-carnitine decreases myocardial injury in children undergoing open-heart surgery: A randomized controlled trial

**DOI:** 10.1007/s00431-024-05534-2

**Published:** 2024-04-03

**Authors:** Wael El Feky, Dalia El-Afify, Dina Abdelhai, Mohamed Elkashlan, Ahmed Fakhreldin, Doaa El Amrousy

**Affiliations:** 1 Cardiothoracic Surgery Department, Faculty of Medicine, Kafr Elsheikh University, Kafr Elsheikh, Egypt; 2 Clinical Pharmacy Department, Faculty of Pharmacy, Tanta University, Tanta, Egypt; 3 Clinical Pathology Department, Faculty of Medicine, Tanta University, Tanta, Egypt; 4 Anesthesia Department, Elmenshawy Hospital, Ministry of Health, Tanta, Egypt; 5 Pediatric Department, Faculty of Medicine, Aswan University, Aswan, Egypt; 6 Pediatric Department, Faculty of Medicine, Tanta University, Tanta, Egypt

**Keywords:** L-carnitine, Children, Open-heart surgery, Oxidative stress, Apoptosis

## Abstract

Myocardial injury in open-heart surgery is related to several factors including ischemia–reperfusion injury, generation of reactive oxygen species, increased production of inflammatory mediators, and enhancement of apoptosis of cardiomyocytes. The aim of this study was to study the effect of L-carnitine on myocardial injury in children undergoing open-heart surgery. This clinical trial was performed on 60 children with congenital heart disease (CHD) who underwent open-heart surgery. They were randomized into two groups: L-carnitine group who received L-carnitine 50 mg\kg\day once daily for 1 month before cardiac surgery and control group who received placebo for 1 month before cardiac surgery. Left ventricular cardiac function was assessed by conventional echocardiography to measure left ventricular ejection fraction (LVEF) and two-dimensional speckle tracking echocardiography (2D-STE) to determine left ventricular global longitudinal strain (2D-LV GLS). Blood samples were obtained pre-operatively at baseline before the administration of L-carnitine or placebo and 12 h post-operatively to measure the level of malondialdehyde (MDA), superoxide dismutase (SOD), fas, caspase-3, creatinine kinase-MB (CK-MB), and troponin I. L-carnitine group had significantly lower post-operative level of oxidative stress marker (MDA), apoptosis markers (fas and caspase-3), and myocardial injury markers (CK-MB and troponin I), but they had significantly higher SOD post-operative level compared to the control group. In addition, post-operative LVEF and 2D-LVGLS were significantly lower in the control group compared to L-carnitine group.

*  Conclusion*: L-carnitine can reduce myocardial injury, improve post-operative left ventricular cardiac function, and may provide myocardium protection in children with CHD who underwent open-heart surgery.

*  Trial registration*: The clinical trial was registered at www.pactr.org with registration number PACTR202010570607420 at 29/10/2020 before recruiting the patients.

**Table:** 

**What is Known:** *• Myocardial injury in open-heart surgery is related to several factors including ischemia–reperfusion injury, generation of reactive oxygen species, increased production of inflammatory mediators, and enhancement of apoptosis of cardiomyocytes.* *• L-carnitine was reported to have myocardial protective effects in rheumatic valvular surgery and coronary artery bypass graft (CABG) in adults; however, there is no evidence on its effectiveness in children undergoing open-heart surgery.*	
**What is New:** *• L-carnitine significantly lowered the post-operative level of oxidative stress marker (MDA), apoptosis markers (fas and caspase-3), and myocardial injury markers (CK-MB and troponin I) in the treatment group.* *• L-carnitine can reduce myocardial injury, improve post-operative left ventricular cardiac function, and may provide myocardium protection in children with CHD who underwent open-heart surgery.*

**Supplementary Information:**

The online version contains supplementary material available at 10.1007/s00431-024-05534-2.

## Introduction

Myocardial injury and myocardial dysfunction after cardiac surgery for congenital heart disease (CHD) are closely related to morbidity and mortality [1]. Cardiopulmonary bypass (CPB) is related to multiple effects that may contribute to post-operative myocardial injury and cardiac dysfunction, including ischemia–reperfusion injury after removal of the aortic clamp, the subsequent generation of reactive oxygen species, and increased production of inflammatory mediators [2, 3]. The pediatric heart especially in neonates has immature antioxidant defenses and is more susceptible to oxidative stress than the adult myocardium [4, 5]. Oxidative stress may promote myocardial injury either due to direct myocardial damage through lipid peroxidation and protein changes or through the induction of apoptosis in cardiomyocytes that are exposed to ischemia–reperfusion [6, 7]. There are two apoptotic pathways: the extrinsic pathway, which is activated by death receptors, and the intrinsic pathway, which can be activated by hypoxia and oxidative stress that occur due to cardiopulmonary bypass [8, 9].

Fas is a member of the death receptor family that induces apoptosis through binding to the Fas ligand. Fas is expressed in different cells, including cardiomyocytes, immune system cells, and the vascular endothelium [10]. Apoptosis involves the activation of a group of proteases called caspases. Caspase 3 plays a central role in apoptosis, either initiated through the extrinsic or intrinsic pathways and is considered a marker of cardiomyocyte apoptosis after open-heart surgery [11].

L-carnitine is an endogenous amino acid that has an important role in myocardial fatty acid metabolism and energy production [12]. It was reported to improve cardiac function in patients with chronic heart failure and dilated cardiomyopathy [13, 14] and to have myocardial protective effects in rheumatic valvular surgery and coronary artery bypass graft (CABG) in adults [15–18]. 

This study aimed to investigate the possible myocardial protective effect of L-carnitine in children undergoing open-heart surgery by investigating L-carnitine effects on oxidative stress, apoptosis, and myocardial injury induced by cardiopulmonary bypass.

## Methods

This randomized controlled clinical trial was performed on 60 children with CHD who underwent open-heart surgery at cardiothoracic surgery departments, Tanta and Kafr ElSheikh University Hospitals, after the approval of the research ethics committee of the Faculty of Medicine, Tanta University. Written consent was obtained from the parents of all children included in this study. The clinical trial was registered at www.pactr.org with ID: PACTR202010570607420 before recruiting the patients.

Inclusion criteria were children aged 2 years or more undergoing open-heart surgery to repair CHD.

Exclusion criteria were patients with metabolic disorders, infective endocarditis, heart failure, children with renal and hepatic diseases, and the administration of drugs with anti-inflammatory or antioxidant effects.

We randomized patients into two groups:

- L-carnitine group: included 30 patients who received 50 mg/kg/day once daily for one month before the cardiac surgery.
- The control group: included 30 patients who received a placebo in the form of glucose 5% for one month before the cardiac surgery.

Patients were randomized to the study groups using simple randomization through a randomization table created by a computer software program. The randomization was carried out by an independent statistician. Allocation concealment was performed using sealed opaque envelopes with sequential numbers. After signing the consent, the sealed opaque envelope was opened, and the patient was enrolled in the respective group.

Left ventricular cardiac function was measured in all patients pre-operatively at baseline before the administration of L-carnitine or placebo and 12 h post-operatively using conventional echocardiography to assess left ventricular ejection fraction (LVEF) and two-dimensional speckle tracking echocardiography (2D-STE) to determine left ventricular global longitudinal strain (2D-LV GLS). According to our protocol of post-operative management, weaning from ventilation and sedatives occurred in the first 2–4 h post-operatively depending on the patients’ condition. So, ventilation and sedatives would not affect echocardiographic examination results.

Venous blood samples were obtained from all patients pre-operatively at baseline before the administration of treatment and 12 h post-operatively to measure the following biochemical parameters:

1. Oxidative stress markers
  -Serum malondialdehyde (MDA): the lipid peroxidation marker that reflects the oxidative stress status was measured using the thiobarbituric acid reactive substances (TBARS) method using a commercial kit (Biodiagnostic, Egypt).
  -Superoxide dismutase (SOD) activity: SOD is an enzyme that catalyzes the dismutation of the superoxide radical into ordinary molecular oxygen and hydrogen peroxide. It was measured using a commercial kit (Biodiagnostic, Egypt).
2. Apoptosis markers
  Serum soluble Fas and serum caspase-3 were determined using commercial enzyme-linked immunosorbent assay (ELISA) kits (Raybiotech Inc, USA).
3. Cardiac markers
  Serum creatine kinase-MB (CK-MB) was determined by the kinetic method using commercial kits (Spectrum, Egypt), and troponin I was measured using a commercial ELISA kit (DRG International Inc., USA).

Post-operative need for inotropes, occurrence of arrhythmias, and mortality were recorded for both groups. The primary outcome was to evaluate troponin I levels 12 h after cardiac surgery. The secondary outcomes were to assess LVEF, 2D-LV GLS, CK-MB, MDA, SOD, fas, and caspase-3 levels 12 h after cardiac surgery.

## Statistical analysis

A power analysis was carried out using the G Power 3.1 program. The sample size of 30 patients in each group was required to achieve a power of 90% with alpha = 0.05 to detect a medium to large effect size of 0.86 in post-operative troponin I level. Data were analyzed using SPSS software version 23 (SPSS Inc. Chicago, IL, USA). Continuous quantitative data were presented as mean ± standard deviation (SD). Qualitative data were presented as numbers and percentages and compared using the chi-square test (*X*^2^). A Student’s *t*-test was used for comparing the means of the quantitative data in the two groups. A paired *t*-test was used to assess significant differences within each studied group before and after open-heart surgery. *P*-value of less than 0.05 was considered statistically significant.

## Results

**Fig. 1:**
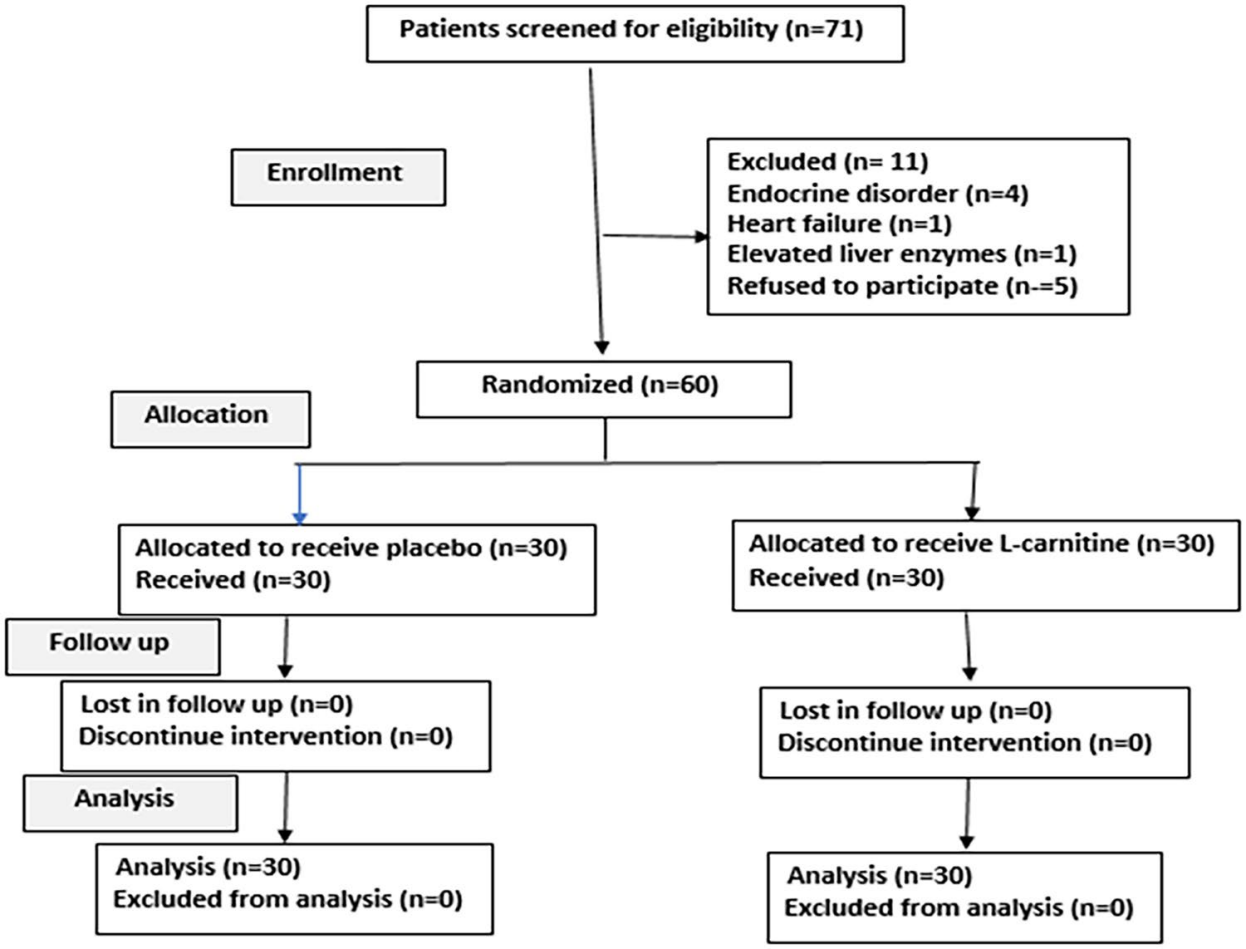
Flow chart of the study showing details of the enrollment, allocation, follow-up, and analysis during the study

The study included 60 children with CHD, 35 patients with atrial septal defect (ASD), and 25 patients with ventricular septal defect (VSD) who underwent open-heart surgery. Their mean age was 3.8 ± 1.2 years and included 26 males and 34 females (flow chart is shown in Fig. 1). The pre-operative clinical data and operative data of the control and L-carnitine groups are presented in Table 1.

**Table 1:** The demographic, pre-operative echocardiographic and laboratory data, and operative data in both groups

Parameter	Control group	L-carnitine group	*p*-value
Age (years)	3.8 ± 1.2	3.9 ± 1.3	0.835
Sex M:F	12\18	14\16	0.602
ASD *n* (%)	16 (53%)	19 (63%)	0.432
VSD *n* (%)	14 (47%)	11 (37%)	
LVEF (%)	68.7 ± 3.2	67.5 ± 3	0.8
2D-LV GLS	− 20.1 ± 2.2	− 19.7 ± 2.1	0.434
CK-MB (UL/L)	5.2 ± 0.69	5 ± 0.71	0.286
Troponin I (ng/L)	0.1 ± 0.06	0.11 ± 0.05	0.927
MDA (nmol/ml)	3.6 ± 1.14	3.8 ± 1.15	0.586
SOD (U/ml)	4.5 ± 0.8	4.7 ± 0.75	0.294
Fas (pg/ml)	63.6 ± 8.4	66.6 ± 9.2	0.189
Caspase-3 (ng/ml)	0.36 ± 0.06	0.37 ± 0.05	0.544
Aortic clamp duration (min)	36.3 ± 8.9	37.4 ± 6.9	0.583
Cardiopulmonary bypass time (min)	51 ± 8.7	52 ± 6.7	0.618

*ASD* atrial septal defect, *VSD* ventricular septal defect, *LVEF* left ventricular ejection fraction, *2D-LVGLS* two-dimensional left ventricular global longitudinal strain, *CK-MB* creatinine kinase-MB, *MDA* malondialdehyde, *SOD* superoxide dismutase

There was no significant difference between the control group and the L-carnitine group regarding age, sex, or the type of open-heart surgery. In addition, the pre-operative CK-MB and troponin I levels were not significantly different between the control and L-carnitine groups. The pre-operative level of the oxidative stress markers (MDA and SOD) and the apoptosis markers (fas and caspase-3) also did not show any significant difference between the control and L-carnitine groups. Moreover, there was no significant difference in pre-operative LVEF or 2D-LV GLS between both groups. Concerning the surgical procedure that was carried out for patients in the two studied groups, the aortic clamp duration and the cardiopulmonary bypass time were more or less comparable in both groups.

There was a significant increase in post-operative serum levels of the cardiac markers (CK-MB and troponin I), oxidative stress markers (MDA), and apoptosis markers (fas and caspase-3) but a significant decrease in post-operative SOD compared to their respective pre-operative levels in each of the control and L-carnitine groups (*p* < 0.001), as shown in Table 2. However, the post-operative serum levels of CK-MB, troponin I, MDA, fas, and caspase-3 in the L-carnitine group were significantly lower than those of the control group, but the post-operative SOD levels in the L-carnitine were significantly higher compared to that of the control group, as shown in Table 2. The post-operative LVEF and 2D-LVGLS significantly decreased in the control group but they were comparable to their respective baseline pre-operative values in L-carnitine group. However, post-operative LVEF and 2D-LV GLS were significantly higher in patients who received L-carnitine compared to the control group. No side effects from L-carnitine were observed in the treatment group.

**Table 2:** Changes in left ventricular cardiac function, oxidative stress, apoptotic, and cardiac markers before and after cardiac surgery in both groups

Parameter	Control group		L-carnitine group		*p*-value
	Pre-operative	Post-operative	Pre-operative	Post-operative	
Troponin I (ng/ml)	0.1 ± 0.06	1.19 ± 0.2^a^	0.11 ± 0.05	0.58 ± 0.13^a^	0.001
CK-MB (U/L)	5.2 ± 0.69	43 ± 6.6^a^	5 ± 0.71	25.3 ± 6.2^a^	0.002
MDA (nmol/ml)	3.6 ± 1.14	5.4 ± 1.1^a^	3.8 ± 1.15	4.1 ± 1.2^a^	0.004
SOD (U/ml)	4.5 ± 0.8	3.2 ± 0.83^a^	4.7 ± 0.75	3.9 ± 0.7^a^	< 0.001
Fas (pg/ml)	63.6 ± 8.4	97 ± 11.5^a^	66.6 ± 9.2	87.7 ± 10.3^a^	0.002
Caspase-3 (ng/ml)	0.36 ± 0.06	0.55 ± 0.09^a^	0.37 ± 0.05	0.42 ± 0.06^a^	0.001
LVEF (%)	68.7 ± 3.2	57.8 ± 4.3^a^	67.5 ± 3	63.5 ± 3.5	0.03
2D-LVGLS	− 20.1 ± 2.2	− 15.7 ± 2^a^	− 19.7 ± 2.1	− 18.1 ± 1.89	0.009

*LVEF* left ventricular ejection fraction, *2D-LVGLS* two-dimensional left ventricular global longitudinal strain, *CK-MB* creatinine kinase-MB, *MDA* malondialdehyde, *SOD* superoxide dismutase. *P* difference between mean post-operative value in the control group and L-carnitine group

^a^Post-operative mean value was significantly different from its respective pre-operative mean values within the same group (*p* ≤ .05)

Post-operative need for inotropic support, incidence of arrhythmias, and mortality were lower in L-carnitine group compared to the control group but none of which reach a significant level (Table 3).

**Table 3:** Post-operative variables in both groups

Parameter	Control group (*N* = 30)	L-carnitine group (*N*-30)	*p*-value
Need for inotropes *N* (%)	4 (13.3%)	2 (6.7%)	0.389
Arrhythmias *N* (%)	3 (10%)	1 (3.3%)	0.301
Mortality *N* (%)	1 (3.3%)	0 (0%)	0.313

## Discussion

Apoptosis in cardiomyocytes that have been subjected to ischemia and reperfusion in open-heart surgery is associated with post-operative myocardial dysfunction, and myocardial stunning and prevention of cardiac apoptosis could provide myocardial protection in open-heart surgery in both children and adults [19]. In this study, we investigated, for the first time, the possible effect of L-carnitine on myocardial injury in children undergoing open-heart surgery for CHD.

L-carnitine caused a significant reduction in the level of post-operative apoptosis markers (fas and caspase-3) and post-operative oxidative stress markers (MDA), a significant increase in post-operative SOD. In addition, it resulted in a significant reduction in cardiac enzymes (CK-MB and troponin I) compared to the control group, which indicates that L-carnitine could provide myocardial protection in pediatric cardiac surgery.

Apoptosis is a programmed process of cell death for the removal of injured cells, and it can be triggered in open-heart surgery by various factors, including myocardial stretch, increased inflammatory cytokines production, and oxidative stress during cardioplegia that involves myocardial ischemia and reperfusion [20]. Our results revealed a significant increase in the post-operative level of apoptosis markers (fas and caspase-3) in both groups compared to their pre-operative respective data. These results are consistent with the results of other studies [21–23].

In our study, L-carnitine administration significantly reduced the post-operative level of the apoptotic cell-death markers (fas and caspase-3) compared to the control group, which is in agreement with the results of Li et al. [15] and Xiang et al. [18], who reported that L-carnitine reduces cardiopulmonary bypass induced cardiomyocyte apoptosis in patients undergoing valve replacement cardiac surgery. L-carnitine may exert its anti-apoptotic effect by different mechanisms, including stabilization of the mitochondrial membrane of cardiac cells and inhibition of cytochrome-C release from the mitochondria. It also inhibits fas-induced apoptosis and inhibits caspase cleavage and activation [24, 25].

In the present study, there was a significant increase in the post-operative level of the oxidative stress marker MDA but a significant decrease in the SOD antioxidant enzyme in patients of both groups compared to their baseline values, which is in agreement with other studies that reported an increase in oxidative stress after pediatric open-heart surgery [26, 27]. This increase in oxidative stress is due to an increase in the production of reactive oxygen species due to cardiopulmonary bypass and the subsequent ischemia–reperfusion injury. L-carnitine significantly decreased the post-operative MDA level but significantly increased the post-operative SOD level compared to that of the control group. This antioxidant effect of L-carnitine was previously reported in patients who underwent rheumatic valve replacement surgery [28] and may be explained by the ability of L-carnitine to activate nuclear factor erythroid 2-related factor 2 (Nrf2); the transcription factor that regulates the expression of antioxidant enzymes [28, 29], and the ability of L-carnitine to reduce the production of reactive oxygen species [30]. The antioxidant effect of L-carnitine can also reduce oxidative stress-induced apoptosis and may contribute to its anti-apoptotic effect.

Troponin I and CK-MB are the most important diagnostic markers for myocardial injury [31], and they significantly increased after pediatric open-heart surgery [32, 33]. This is consistent with our results of the post-operative increase of CK-MB and troponin I in both the control and L-carnitine groups. The L-carnitine group had a significantly lower level of post-operative CK-MB and troponin I compared to the control group, and this is in agreement with other previous studies [15, 16, 18].

In addition, the L-carnitine group had significantly higher post-operative LVEF and 2D-LVGLS compared to the control group, which reveals that L-carnitine administration improves post-operative left ventricular cardiac function, which was also observed in other previous studies [16, 18, 34]. These results suggest that L-carnitine can decrease myocardial injury and improve left ventricular cardiac function after open-heart surgery in children with CHD.

## Study limitations

The study included a small sample size and a relatively short duration of follow-up. Further studies on a larger scale, including patients with more complex congenital heart diseases, using different doses and duration of treatment of L-carnitine are recommended with an assessment of the L-carnitine effect on morbidity and mortality.

## Conclusion

L-carnitine can reduce myocardial injury, improve post-operative left ventricular function, and may provide myocardium protection in children with congenital heart disease during open-heart surgery through exerting anti-apoptotic and antioxidant effects.

### Supplementary Information

Below is the link to the electronic supplementary material.

Supplementary file1 (DOC 217 KB)

## Data Availability

The dataset used and/or analyzed during the current study is available from the corresponding author on reasonable request.

## Abbreviations

ASD: Atrial septal defect
CBP: Cardiopulmonary bypass
CHD: Congenital heart disease
CK-MB: Creatine kinase-MB
ELISA: Enzyme-linked immunosorbent assay
LVEF: Left ventricular ejection fraction
MDA: Malondialdehyde
SD: Standard deviation
SOD: Superoxide dismutase
TBARS: Thiobarbituric acid reactive substances
VSD: Ventricular septal defect
2D-LV GLS: Two-dimensional left ventricular global longitudinal strain
2D-STE: Two-dimensional speckle tracking echocardiography
